# Transferring clinically established immune inflammation markers into exercise physiology: focus on neutrophil-to-lymphocyte ratio, platelet-to-lymphocyte ratio and systemic immune-inflammation index

**DOI:** 10.1007/s00421-021-04668-7

**Published:** 2021-03-31

**Authors:** David Walzik, Niklas Joisten, Jonas Zacher, Philipp Zimmer

**Affiliations:** 1 Institute for Sport and Sport Science, TU Dortmund University, Dortmund, Germany; 2 Institute for Cardiovascular Research and Sport Medicine, German Sport University Cologne, Cologne, Germany

**Keywords:** Physical activity, Exercise, Training, Recovery, Inflammation, Biomarker

## Abstract

Over the last decades the cellular immune inflammation markers neutrophil-to-lymphocyte ratio (NLR), platelet-to-lymphocyte ratio (PLR) and systemic immune-inflammation index (SII = NLR × platelets) have emerged in clinical context as markers of disease-related inflammation and are now widely appreciated due to their integrative character. Transferring these clinically established inflammation markers into exercise physiology seems highly beneficial, especially due to the low temporal, financial and infrastructural resources needed for assessment and calculation. Therefore, the aim of this review is to summarize evidence on the value of the integrative inflammation markers NLR, PLR and SII for depiction of exercise-induced inflammation and highlight potential applications in exercise settings. Despite sparse evidence, multiple investigations revealed responsiveness of the markers to acute and chronic exercise, thereby opening promising avenues in the field of exercise physiology. In performance settings, they might help to infer information for exercise programming by reflecting exercise strain and recovery status or periods of overtraining and increased infection risk. In health settings, application involves the depiction of anti-inflammatory effects of chronic exercise in patients exhibiting chronic inflammation. Further research should, therefore, focus on establishing reference values for these integrative markers in athletes at rest, assess the kinetics and reliability in response to different exercise modalities and implement the markers into clinical exercise trials to depict anti-inflammatory effects of chronic exercise in different patient collectives.

## Introduction

Acute inflammation is a physiological response of the human body to local tissue damage, aiming at restoring tissue integrity and tissue homeostasis in both physiological context such as exercise (Chazaud 2016) and pathological context such as disease (Eming et al. 2017). Although this response is essential for long-term health, it is often accompanied by negatively perceived side effects such as swelling, pain, heat, redness and impaired function, which are mainly mediated by vasodilation and increased perfusion. Beside this vascular response, a cellular response is initiated by the immune system to identify and eliminate the inflammatory triggers and remove damaged tissue (Medzhitov 2008). Since tissue damage can occur in response to different stimuli, the connotation of acute inflammation is context dependent. In clinical context a bacterial infection might cause acute inflammation and even provoke severe complications, while this is rarely the case in response to mechanical tissue damage, as induced in skeletal muscle by strenuous exercise. Here, acute inflammation represents a transient state, which can be seen as the first step of the subsequent recovery process (Peake et al. 2017). In both cases inflammation aims at restoring tissue function and usually resolves as soon as homeostasis is re-established (Eming et al. 2017). However, if inflammatory processes do not result in restoration of homeostasis, acute inflammation remains unresolved, ultimately resulting in chronic inflammation, a hallmark and central risk factor of various medical conditions (Nathan and Ding 2010). In this context, attention has been drawn to the potential benefit of chronic exercise interventions. While acute exercise induces an inflammatory response, chronic exercise has shown to exert anti-inflammatory effects via several mechanisms, thereby bearing strong potential in the prevention and treatment of diseases linked to chronic inflammation (Gleeson et al. 2011).

Due to the fact that acute inflammation occurs in both clinical and exercise-based settings, it is no surprise to see a rising number of clinical inflammation markers such as C-reactive protein (CRP) or interleukin-6 (IL-6) find their way into exercise physiology. These inflammation markers are usually assessed as objective correlates of inflammatory processes to give an insight into the individual inflammatory state, thereby allowing predictions as to *when* and *how* the next exercise session can be carried out (Pedlar et al. 2019). Building upon this, the aim of the present review is to assess the potential value of the novel but clinically established cellular immune inflammation markers neutrophil-to-lymphocyte ratio (NLR), platelet-to-lymphocyte ratio (PLR) and systemic immune-inflammation index (SII = NLR × platelets) in the field of exercise physiology. After focusing on strengths and limitations of some of the most frequently used inflammation markers in exercise settings, we seek to highlight the benefits and potential applications of the calculated markers NLR, PLR and SII in performance- and health-related exercise settings. By providing a detailed description on how to interpret these markers we want to emphasize their practical relevance and encourage future exercise research to implement them in both clinical intervention trials and elite sport settings.

## Inflammation markers—from clinical context to exercise physiology

When considering blood-derived inflammation markers, it is important to keep in mind that they mostly originate from clinical settings. Inflammatory processes, patient characteristics and (patho) physiology can differ considerably compared to exercise settings, thereby potentially changing the way of interpretation. Nonetheless, inflammation markers are of great benefit in exercise settings since they allow an objective and physiology-based insight into the individual inflammatory state. Beside basic research approaches investigating the physiology behind acute and chronic exercise-induced alterations of immune homeostasis in different populations, further application includes performance and health settings. In performance settings, inflammation markers are frequently used to measure exercise strain and recovery processes, as indicated by altered values in response to acute exercise. Return to baseline of these markers is interpreted as restoration of homeostasis, suggesting completion of the recovery process. Reflecting individual recovery kinetics, inflammation markers are, therefore, assessed to improve exercise programming by adjusting exercise characteristics (e.g., frequency, intensity, type, time) to the individual recovery state. However, prior correlation of the respective inflammation markers with performance measures is crucial to ensure validity. Apart of post-exercise deviations, altered baseline concentrations of several biomarkers have additionally been suspected to depict periods of overtraining or increased susceptibility to infection (Gleeson 2002; Lee et al. 2017). In health settings, inflammation markers are assessed to reflect anti-inflammatory properties of chronic exercise in different pathologies. More precisely, baseline inflammation markers have been shown to decrease as a consequence of regular exercise training, especially in medical conditions linked to chronic inflammation, e.g. cardiovascular and neoplastic diseases (Gleeson et al. 2011; Ortega 2016). In view of these benefits, blood-derived inflammation markers are increasingly assessed in both performance and health-related exercise settings. However, due to numerous markers with potential application (Lee et al. 2017; Reichel et al. 2020), only some of the frequently assessed are presented in the following paragraphs.

A hallmark of exercise is the mechanical strain on muscle tissue. Therefore, muscle-derived damage markers have received considerable research attention as indicators of exercise stress and recovery process in the past decades. With creatine kinase (CK) one of these markers was first applied in exercise context as early as 1965 (Vejjajiva and Teasdale 1965). While originally used as a biomarker in clinical conditions linked to muscle tissue damage (e.g., myocardial infarction, myopathies), CK is nowadays frequently assessed in exercise context as well. As a key enzyme in ATP regeneration, it is suspected to leak into peripheral blood via the lymphatic system after microtrauma in skeletal muscle, mostly induced by high mechanical strain on muscle tissue (e.g., eccentric exercise). An interesting alternative hypothesis suspects *volitional* expulsion of CK in metabolically stressed cells to avoid cell death (Behringer et al. 2014). Although correlation of CK concentrations with performance parameters was demonstrated in several investigations (Baird et al. 2012), its usefulness as a biomarker in exercise settings is discussed controversially due to several limitations. Firstly, there is a great interindividual variability in dependence on subject characteristics (e.g., sex, ethnicity, age, muscle mass), making determination of individual reference values a crucial step for frequent assessment. Secondly, values differ depending on the applied exercise stimulus, with the highest values occurring after exercise modalities related to a high amount of muscle damage (e.g., prolonged and eccentric exercise), thereby limiting applicability in other exercise settings (Brancaccio et al. 2007). Thirdly, there also seem to be differences in CK-response depending on training status, leading to distinction of *high-responders* and *low-responders* (Vincent and Vincent 1997). A recent investigation on the reliability of different recovery biomarkers revealed a moderate reliability of CK in response to acute aerobic exercise. Interestingly, when accounting for training status, reliability was poor in the trained subgroup, thereby further complicating the use of CK as muscle damage biomarker, especially in athletes, where it is most frequently assessed (Reichel et al. 2020). However, it is important to separate exercise-induced muscle damage from inflammation as such. While CK—though characterized by several limitations—is a biomarker of muscle damage, its role in classic inflammatory processes is rather inferior.

When looking for markers that depict exercise-induced inflammation, immunological parameters represent an easily accessible physiological resource. Due to the strong involvement of the immune system in inflammatory processes, several components of both the humoral and cellular compartment have been considered as inflammation markers in exercise settings (Gonçalves et al. 2020). As part of the humoral compartment acute phase reactants such as CRP are frequently assessed. Similar to CK, its use in exercise settings originates from a clinical background, where CRP is used as a general marker of inflammation in a broad range of diseases (Luan and Yao 2018). While CRP levels generally increase in response to acute exercise, baseline levels seem to decrease in response to chronic training, thereby reflecting the (anti-)inflammatory effects of acute and chronic exercise (Kasapis and Thompson 2005). Aside of CRP, another frequently applied inflammation marker is the inflammatory cytokine IL-6, which is substantially involved in the innate and adaptive immune response, e.g. via the production of acute phase proteins and the proliferation of T- and B-cells (Van Snick 1990). Serving as a general marker of inflammation in clinical context it was transferred to exercise settings after discovering that peripheral IL-6 concentrations increase in response to acute exercise. In fact, skeletal muscle contractions themselves are responsible for the majority of exercise-induced increases in IL-6, making it an attractive biomarker to depict exercise-induced inflammation (Fischer 2006). However, for both CRP and IL-6 only moderate reliability was found in response to acute aerobic exercise, thereby questioning their benefit in exercise settings (Reichel et al. 2020). Additionally, the high methodological resources needed for determination render a frequent assessment impractical.

Finally, exercise-induced inflammation is also reflected by the cellular compartment of the immune system. Since immunological alterations are part of any inflammatory reaction, immune cells are an interesting target in exercise context. Although acute exercise generally induces a strong leukocytosis, kinetics of leukocyte subsets can differ considerably. For instance, neutrophil and lymphocyte counts increase during exercise (neutrophilia, lymphocytosis), but show different post-exercise kinetics. The response of neutrophils is marked by a persistent neutrophilia, while lymphocyte counts decrease within 10–15 min after exercise cessation (lymphocytopenia) (Shek et al. 1995; Pedersen et al. 1998). Mechanistically, neutrophilia and lymphocytosis are explained by mobilization of marginal immune cell pools in the liver, spleen, lung and on vessel walls via the action of catecholamines and increased shear stress mediated by higher perfusion (Simpson et al. 2015). The persistent neutrophilia is additionally promoted by a cortisol-induced release of neutrophils from the bone marrow (Yamada et al. 2000). Beside affecting the absolute number of neutrophils and lymphocytes, catecholamines and glucocorticoids also impact immune cell function (Simpson et al. 2015) and act differently in healthy individuals compared to diseased (McMurray and Hackney 2005; Ortega 2016). While the mechanisms behind neutrophilia and lymphocytosis are fairly well understood, post-exercise lymphocytopenia is interpreted differentially. One hypothesis suspects an impaired immune function due to apoptosis of lymphocytes after acute strenuous bouts of exercise (Kakanis et al. 2010), while another hypothesis assumes emigration of lymphocytes from the circulation to peripheral tissue, thereby increasing immune competence and surveillance (Campbell and Turner 2018). Whether acute exercise causes an increase or decrease in infection risk of healthy individuals remains inconclusive until today and is still a topic of hot debate (Simpson et al. 2020). However, independent of the immunological consequences, leukocyte count seems to represent a useful physiological correlate of inflammatory processes, as reinforced by numerous investigations in exercise settings (Peake et al. 2005; Cerqueira et al. 2020).

## Introducing the cellular immune inflammation markers NLR, PLR and SII

Although total leukocyte count is a useful measure to depict general inflammation, it fails to consider the distinct kinetics of different leukocyte subsets. Tackling this problem, the integrative cellular immune inflammation markers NLR, PLR and SII have emerged in clinical context during the past decades. Considering multiple immune cell populations, they provide a multifactorial insight into inflammatory processes. Although no conclusions can be drawn on the kinetics of lymphocyte subsets such as T- and B-cells, these markers are increasingly implemented as inflammatory and prognostic markers in various clinical conditions such as neoplastic (Howard et al. 2019; Yang et al. 2020), neurological (Hemond et al. 2019) or cardiovascular diseases (Bhat et al. 2013). Moderate to high correlations between these markers and well-established inflammation markers such as white blood cell count (Gonda et al. 2017), CRP (Huang et al. 2018; Quartuccio et al. 2020), IL-6 (Islas-Vazquez et al. 2020; Zhu et al. 2020) and erythrocyte sedimentation rate (Huang et al. 2018) additionally underline the suitability for depiction of inflammatory processes. Surprisingly, application of these markers in exercise settings is sparse so far. Since the integrative value of the markers is exploited in clinical context to adapt therapeutic measures to the patient’s inflammatory status (Cai et al. 2021), conclusions in exercise settings could be drawn in a similar manner, e.g. to custom exercise programs to the individual recovery needs. Considering these potential benefits, we seek to introduce the clinically established cellular immune inflammation markers NLR, PLR and SII in the field of exercise physiology, thereby highlighting their potential value in depiction of exercise-induced inflammation.

**Fig. 1:**
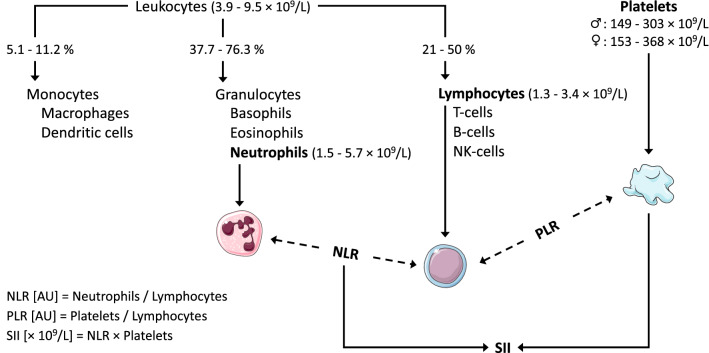
Calculation of the cellular immune inflammation markers neutrophil-to-lymphocyte ratio (NLR), platelet-to-lymphocyte ratio (PLR) and systemic immune-inflammation index (SII). Altered concentrations of the underlying blood cell populations in response to acute exercise are represented by different height, with higher placement indicating higher concentrations. Dashed lines indicate division of blood cell populations; solid lines indicate multiplication. Numeric reference values were extracted from Arbiol-Roca et al. (2018)

As a calculated ratio of leukocyte subsets the NLR was first proposed as an inflammatory marker after observing that cancer patients exhibit sustained neutrophilia accompanied by lymphocytopenia (Zahorec 2001). Since then, numerous studies have investigated the value of NRL as an inflammatory and prognostic marker in cancer settings (Guthrie et al. 2013) and other diseases (Bhat et al. 2013; Okyay et al. 2013). Interestingly, the NLR has also been studied in the context of exercise as early as 1995 (Nieman et al. 1995). Since sustained neutrophilia and lymphocytopenia are also characteristic for the early recovery stages after exercise, transfer of the NLR as an acute inflammation marker seems reasonable. However, only few studies have assessed this potential value in the context of exercise since then (see section “Current state of knowledge—NLR, PLR and SII in exercise physiology”). By integrating the kinetics of the two largest leukocyte subsets into one condensed parameter, NLR seems to have high potential as an inflammation marker in exercise settings with increased values indicating ongoing inflammatory processes. Considering the kinetics of NLR, the highest values arise when neutrophils counts are high and lymphocytes counts are low (see Fig. 1).

A second cellular immune inflammation marker is the PLR. In contrast to NLR, this marker is not only based on leukocyte subsets, but takes platelet counts into consideration. Beside the well-known role of platelets in primary haemostasis, they also exhibit various pro-inflammatory properties, underlining their value as inflammation marker (Zarbock et al. 2007). Similar to NLR, current research has mainly focused on diseased populations, establishing both NLR and PLR as inflammation markers in diseases such as cancer (Stojkovic Lalosevic et al. 2019). In some medical conditions (e.g., renal disease) PLR was even found to be a better marker of disease-related inflammation than NLR (Turkmen et al. 2013), thereby raising interesting questions as to which marker proves more beneficial. Surprisingly, PLR has thus far found very little consideration in the context of exercise (see section “Current state of knowledge—NLR, PLR and SII in exercise physiology”), most likely due to the combination of two apparently distinct blood cell populations. Similar to exercise-induced neutrophilia, platelet counts rise acutely in response to exercise (thrombocytosis) due to a fresh release from the bone marrow, spleen and pulmonary intravascular pools (El-Sayed et al. 2000). Therefore, PLR can be seen as an alternative to NLR, replacing neutrophils with platelets in the calculation of the cellular immune inflammation markers (see Fig. 1). Considering exercise-induced thrombocytosis, PLR seems equally valuable to depict inflammation in response to acute exercise. Similar to NLR highest PLR values arise when platelet counts are high and lymphocyte counts are low.

Only recently, Hu et al. (2014) introduced the SII as a third cellular immune inflammation marker that integrates the kinetics of NLR and PLR into one single parameter. While NLR and PLR are calculated as ratios of two different blood cell populations, respectively, the SII considers three populations by multiplying the NLR with platelet counts (see Fig. 1). In clinical context, the SII has since then gained remarkable popularity as inflammation-based prognostic marker, mainly in cancer settings (Yang et al. 2018). However, to our knowledge there has been no investigations on the potential value of SII in the context of exercise outside of work from our group (see section “Current state of knowledge—NLR, PLR and SII in exercise physiology”). By multiplying NLR values with platelet counts, the effect of exercise-induced neutrophilia and lymphocytopenia (as indicated by NLR) is amplified by the effect of thrombocytosis. Considering various exercise-responsive blood components, SII might constitute a versatile and robust marker in the assessment of exercise-induced inflammation and could represent an alternative or addition to frequently assessed inflammation markers. Similar to NLR, highest SII values occur when neutrophil and platelet counts are high and lymphocyte counts are low (see Fig. 1).

To enable frequent utilization of the integrative markers in clinical context, several authors have determined age- and gender-stratified reference values for NLR, PLR and SII in healthy individuals at rest (see Table 1) (Fest et al. 2018; Meng et al. 2018; Luo et al. 2019). However, since reference values were reported by different descriptive statistics (e.g., mean, median, chosen percentiles), results are hard to compare and seem to vary slightly across the investigations. While the impact of gender was fairly consistent with higher PLR and SII in women and higher NLR in men, the impact of age was reported differently by the authors. Interestingly, Fest et al. (2018) revealed that baseline values of NLR and SII increase with age, which might be attributed to the higher prevalence of inflammation-linked pathologies in elderly. In contrast, PLR decreases with age, which is in accordance with the lower platelet counts found in old individuals (Biino et al. 2013). A further stratification factor that was unfortunately not considered is body mass index. Since body composition can impact baseline values of inflammatory markers substantially, consideration as stratification factor is warranted in future investigations.

**Table 1:** Reference values for NLR, PLR and SII in healthy individuals at rest

Study	Data reported as	NLR [AU]	PLR [AU]	SII [× 10^9^/L]
Fest et al. (2018)	Mean (2.5th, 97.5th percentile)			
Male		1.88 (0.88, 4.14)	112 (57, 230)	453 (185, 1168)
Female		1.68 (0.8, 3.8)	126 (65, 246)	463 (194, 1169)
Aged 45–54		1.63 (0.8, 3.44)	118 (62, 211)	456 (189, 1063)
Aged 55–64		1.61 (0.79, 3.53)	116 (60, 226)	436 (186, 1109)
Aged 65–74		1.82 (0.86, 3.92)	119 (60, 239)	455 (186, 1131)
Aged 75–84		2.02 (0.96, 4.53)	127 (61, 268)	500 (196, 1373)
Aged ≥ 85		2.13 (0.89, 5.86)	131 (63, 282)	522 (205, 1798)
Meng et al. (2018)	Median (25th, 75th percentile)			
Male		1.72 (1.39, 2.17)	102 (85, 124)	358 (275, 466)
Female		1.71 (1.35, 2.18)	115 (95, 140)	374 (282, 497)
Aged 18–65		1.71 (1.36, 2.17)	106 (88, 128)	366 (278, 480)
Aged > 65		1.85 (1.46, 2.36)	139 (116, 169}	366 (275, 488)
Luo et al. (2019)	Median (2.5th, 97.5th percentile)			
Male		1.75 (0.89, 3.95)	94 (46, 181)	329 (142, 764)
Female		1.78 (0.87, 4.06)	105 (51, 206)	341 (141, 850)
Aged 18–64		1.76 (0.88, 4.02)	100 (49, 198)	337 (145, 810)
Aged 65–79		1.81 (0.89, 3.91)	90 (42, 187)	312 (124, 784)

*NLR* Neutrophil-to-lymphocyte ratio, *PLR* Platelet-to-lymphocyte ratio, *SII* Systemic immune-inflammation index, *AU* Arbitrary unit

## Current state of knowledge—NLR, PLR and SII in exercise physiology

To date, evidence regarding the potential value of NLR, PLR and SII as cellular immune inflammation markers for the depiction of exercise-induced inflammation is limited. However, due to the low temporal, financial and infrastructural resources needed for assessment and calculation, application in exercise settings seems highly feasible and easy to implement. Including these markers in both performance- and health-related exercise settings might enable an integrative depiction of exercise-induced inflammation, as reflected by cellular alterations within the bloodstream. In competitive sport these markers might facilitate depiction of exercise strain and recovery processes or help identify periods of increased infection risk or overtraining, thereby improving exercise programming. In health settings, they might indicate anti-inflammatory effects of chronic exercise, especially in patients exhibiting chronic inflammation.

Given these potential applications, the few studies that have investigated NLR, PLR and SII in the context of exercise showed promising results. In response to acute exercise 9 of 11 studies revealed an increase of NLR (see Table 2), suggesting good suitability for depiction of exercise-induced inflammation. Additionally, Joisten et al. (2020) demonstrated an intensity-dependent increase of NLR in response to acute exercise with significantly higher NLR values occurring after acute high intensity interval training compared to moderate continuous training in persons suffering from multiple sclerosis. Comparing the different studies performed on NLR, acute exercise-induced increases can reach up to six-fold above baseline with absolute values over 10 (Nieman et al. 1995). In contrast, chronic exercise interventions seem to show conflicting results at first sight. However, when accounting for the applied exercise intervention, results appear more conclusive. While intensified training periods with the aim of overstressing athletes resulted in an increased baseline NLR (Mackinnon et al. 1997; Svendsen et al. 2016), *physiological* training interventions induced a decrease in both healthy (Makras et al. 2005) and diseased populations (Wang et al. 2011; Joisten et al. 2020). The applicability of NLR as a marker of inflammatory status is further reinforced by the significant correlation of decreased baseline NLR and IL-6 concentrations obtained by Wang et al. (2011) in response to a 4-week exercise and diet intervention in overweight adolescents. Conversely, when exercise activity was decreased, anti-inflammatory effects of chronic exercise quickly diminished. In a study investigating the impact of 8 weeks of detraining on inflammatory status, an increase in NLR by 48.2% was observed (Liao et al. 2016). Additionally, the results of studies investigating intensified training periods indicate a potential application of NLR as marker of chronic exercise overload (Mackinnon et al. 1997; Svendsen et al. 2016). Indeed increased NLR has previously been discussed as a potential immune inflammation marker for impending overtraining (Gleeson 2002).

Compared to NLR, far less evidence is available on the PLR in the context of exercise. Of the five studies investigating the impact of acute exercise on the PLR, three showed increased values post-exercise, indicating an inflammatory response (see Table 2). Considering exercise-induced thrombocytosis instead of neutrophilia, PLR might, therefore, serve as a useful alternative or addition to NLR, as indicated by increases in both markers in response to acute exercise in healthy (Wahl et al. 2020) and diseased populations (Korkmaz et al. 2018). Interestingly, PLR values were altered more profoundly by high intensity exercise modalities with values around twice higher than at rest and absolute values over 200 (Wahl et al. 2020). A possible explanation for this might be the exercise-dependent mobilization of platelets into peripheral circulation (Posthuma et al. 2015). Considering the effect of chronic exercise on the PLR, no alterations were found after 3 weeks of endurance exercise in a population suffering from multiple sclerosis (Joisten et al. 2020). These results seem surprising since persons with multiple sclerosis generally exhibit increased platelet counts associated with chronic inflammation (Dziedzic and Bijak 2019) and chronic exercise has shown to lower inflammatory mediators (Gleeson et al. 2011). As a consequence of this, the baseline PLR would be expected to decrease. However, non-responsiveness of platelets to chronic exercise might be suspected as a potential reason for unaltered values. Regarding the inconsistent results of the few studies conducted so far and the lack of chronic exercise interventions in healthy individuals, there is an urgent need for further research approaches focusing on the potential value of PLR as an exercise-induced inflammation marker in different populations and exercise modalities.

For SII even less evidence is available in the context of exercise. However, three of the four studies conducted so far showed an increased SII after acute exercise, thereby indicating a potentially promising role as inflammation marker in exercise settings (see Table 2). Similar to NLR and PLR, high intensity modalities elicited the most pronounced response with values around three- to four-fold above baseline and absolute values over 1000 (Wahl et al. 2020). Due to the integration of exercise-induced neutrophilia, lymphocytopenia and thrombocytosis, there seems to be a strong physiological basis for the application of SII as inflammation marker in response to acute exercise. Additionally, the SII seems to depict anti-inflammatory effects of chronic exercise in a similar manner as the NLR, as indicated by a decrease in baseline values after 3 weeks of high intensity interval training in persons suffering from multiple sclerosis (Joisten et al. 2020). However, since all investigations on the potential value of SII as inflammation marker in the context of acute or chronic exercise are from our group, replication and extension of our results in future research endeavours is urgently needed. A summary of current studies assessing NLR, PLR and SII in the context of both acute and chronic exercise interventions is provided in Table 2.

**Table 2:** Overview of studies assessing NLR, PLR or SII as inflammation markers in exercise settings

Study	N	Study population	Intervention	NLR	PLR	SII
Acute exercise						
Bessa et al. (2016)	19	Healthy young male cyclists	6x “all out reps” at 85% 1RM for squat and bench press followed by 1 h cycling at 85% VO_2peak_	↑	NA	NA
Davison and Diment (2010)	20	Healthy young males	2 h cycling at 64% VO_2max_	↑	NA	NA
Joisten et al. (2019)	20	Healthy young males	300 countermovement jumps	↑	–	↑
Joisten et al. (2020)^b^	35	Old males and females with MS	HIIT: 5 × 1.5 min cycling at 95–100% HR_max_	-	↑	–
	33		MCT: 24 min cycling at 65% HR_max_	–	–	–
Kerksick et al. (2010)	30	Healthy young males	100 eccentric knee extensions	↑	NA	NA
Korkmaz et al. (2018)	113	Old males and females with symptoms of CAD	Treadmill exercise according to Bruce protocol	↑	↑	NA
Murase et al. (2016)	16	Healthy young males	59 min cycling at 75% VO_2max_	↑	NA	NA
Nieman et al. (1995)^a^	22	Healthy middle-aged male runners	2.5 h running at 75% VO_2max_	↑	NA	NA
Schlagheck et al. (2020)	24	Healthy young males	MCT: 45 min cycling at 60% PPO	–	–	↑
	24		RE: 5 exercise machines, each 4 × 8–10 reps at 70% 1RM	–	–	–
Wahl et al. (2020)	12	Healthy young male triathletes/cyclists	HIIT: 4 × 4 min at 90–95% PPO	↑	↑	↑
			HIIT: 4 × 30 s “all out”	↑	↑	↑
Wei et al. (2017)	12	Healthy young males	Cycling to volitional exhaustion at 75% VO_2max_	–	NA	NA
	9	Healthy middle-aged males	Cycling to volitional exhaustion at 75% VO_2max_	↑	NA	NA
Chronic exercise						
Joisten et al. (2020)^b^	35	Old males and females with MS	3 weeks, 3x/week HIIT: 5 × 1.5 min cycling at 95–100% HR_max_	↓	–	↓
	33		3 weeks 3x/week MCT: 24 min cycling at 65% HR_max_	–	–	–
Mackinnon et al. (1997)	24	Healthy female and male elite swimmers	4 weeks 6x/week twice a day progressive intensified swim training	↑	NA	NA
Makras et al. (2005)	48	Healthy young males	4 weeks 5x/week moderate intermittent mixed EE and RE	↓	NA	NA
Pagola et al. (2020)	13	Old females with breast cancer	16 weeks 2x/week for 75 min intensive mixed EE and RE	–	NA	NA
	10		16 weeks 2x/week unsupervised moderate mixed EE and RE	–	NA	NA
Svendsen et al. (2016)	13	Healthy young male cyclists	8 days of intensified cycling training (increased volume and intensity)	↑	NA	NA
Wang et al. (2011)	43	Obese male adolescents	4 weeks 6x/week twice a day 2 h mixed EE	↓	NA	NA

Significant changes of NLR, PLR and SII are reported as differences from baseline to post-exercise (time effects) to show suitability for depiction of exercise-induced inflammation

*1RM* One-repetition maximum, *CAD* Coronary artery disease, *EE* Endurance exercise, *HIIT* High intensity interval training, *HR*_*max*_ Maximum heart rate, *MCT* Moderate continuous training, *MS* Multiple sclerosis, *NA* Not assessed, *NLR* Neutrophil-to-lymphocyte ratio, *PLR* Platelet-to-lymphocyte ratio, *PPO* Peak power output, *RE* Resistance exercise, *Reps* Repetitions, *SII* Systemic immune-inflammation index, *VO*_*2max*_ Maximal oxygen consumption, *VO*_*2peak*_ Peak oxygen consumption

^↑^Significant increase

^↓^Significant decrease

^–^No significant changes

^a^Results were assessed as differences from a passive control group

^b^Results were obtained in the same study

## Limitations and future perspectives

Considering blood-derived inflammation markers, caution is warranted what conclusions can be drawn from them, since this can differ depending on their physiological origin. Therefore, it is important to stress that NLR, PLR and SII are inflammation markers based on cellular alterations within the bloodstream. In contrast to muscle-derived damage markers such as CK, they allow no assessment of the occurrence of tissue damage or the associated repair processes. Instead, they should be seen as markers of general inflammation in both performance and health-related exercise settings. However, to enable regular assessment, resting values for athletes and kinetics of different exercise modalities have to be established. While already described by several authors in the general population (Fest et al. 2018; Meng et al. 2018; Luo et al. 2019), reference values for athletes and kinetics of different exercise modalities are lacking thus far. Further investigations should, therefore, focus on assessing NLR, PLR and SII in athletes and stratify values by age, gender, training status and exercise modality to determine a baseline range. In this context special consideration should be given to exercise-specific influencing variables such as haematocrit, dietary habits, hydration and hormonal status, since these parameters might influence baseline levels and exercise kinetics.

After establishing baseline values, alterations of these values could be utilized for exercise programming. Regarding the reliability of these markers, a recent study by Reichel et al. (2020) assessed intraclass correlation coefficients between two identical strenuous endurance exercise protocols for several biomarkers, identifying some promising candidates for frequent assessment. Surprisingly, immune cell counts were not altered by the exercise protocol and only moderate reliability was found for NLR, PLR and SII. Since strenuous exercise is known to induce strong immunological alterations, the obtained results seem inconclusive and limit the power of the reliability found for NLR, PLR and SII since their calculation depends directly on immunological alterations. Further research investigating the reliability of the cellular immune inflammation markers in response to different exercise modalities is, therefore, strongly warranted. Although other methodological approaches such as flow cytometry offer a more precise insight into cellular alterations of the immune system in response to exercise, calculation of NLR, PLR and SII is much more feasible and facilitates frequent assessment in practical exercise settings such as competitive sport or rehabilitation programs. Implementing these markers into routine assessments might enable athletes and coaches to infer information on individual recovery needs and help clinical practitioners monitor the anti-inflammatory effects of long-term exercise in patients with chronic inflammation. Since calculation of these markers can be performed with a simple blood count, we strongly encourage future research approaches in exercise physiology to incorporate the presented cellular inflammation markers.

## Conclusion

This review introduced the clinically established cellular immune inflammation markers NLR, PLR and SII in the context of exercise physiology. In response to acute exercise the presented markers might prove beneficial to depict exercise strain and recovery processes in competitive sport. In chronic exercise interventions, they might additionally depict periods of overtraining and increased infection risk in athletes or indicate amelioration of baseline inflammation in patients with chronic inflammation. So far, comparatively few studies have assessed the cellular immune inflammation markers in the context of exercise. Further research should, therefore, focus on establishing reference values for athletes at rest and investigate the kinetics and reliability of the markers in response to different exercise modalities. Since the inflammatory response can differ considerably depending on the applied exercise stimulus and the investigated population, it is crucial to consider these characteristics when assessing inflammation.

## Abbreviations

CK: Creatine kinase
CRP: C-reactive protein
IL-6: Interleukin-6
NLR: Neutrophil-to-lymphocyte ratio
PLR: Platelet-to-lymphocyte ratio
SII: Systemic immune-inflammation index
